# CO_2_ Foamed Viscoelastic Gel-Based Seawater Fracturing Fluid for High-Temperature Wells

**DOI:** 10.3390/gels10120774

**Published:** 2024-11-27

**Authors:** Jawad Al-Darweesh, Murtada Saleh Aljawad, Muhammad Shahzad Kamal, Mohamed Mahmoud, Shabeeb Alajmei, Prasad B. Karadkar, Bader G. Harbi

**Affiliations:** 1 Department of Petroleum Engineering, King Fahd University of Petroleum & Minerals, Dhahran 31261, Saudi Arabia; g200895180@kfupm.edu.sa (J.A.-D.); mmahmoud@kfupm.edu.sa (M.M.); alajmisn@kfupm.edu.sa (S.A.); 2 Center for Integrative Petroleum Research, King Fahd University of Petroleum & Minerals, Dhahran 31261, Saudi Arabia; shahzadmalik@kfupm.edu.sa; 3 EXPECR ARC, Saudi Aramco, Dhahran 31311, Saudi Arabia; prasad.karadkar@aramco.com (P.B.K.); bader.alharbi.1@aramco.com (B.G.H.)

**Keywords:** viscoelastic surfactants, CO_2_ foam, thermally stable, fracturing, proppant transport

## Abstract

This study investigates the development of a novel CO_2_-foamed viscoelastic gel-based fracturing fluid to address the challenges of high-temperature formations. The influence of various parameters, including surfactant type and concentration, gas fraction, shear rate, water salinity, temperature, and pressure, on foam viscosity was systematically explored. Rheological experiments were conducted using a high-pressure/high-temperature (HPHT) rheometer at 150 °C and pressures ranging from 6.89 to 20.68 MPa. To simulate field conditions, synthetic high-salinity water was employed. The thermal stability of the CO_2_ foam was evaluated at a constant shear rate of 100 1/s for 180 min. Additionally, foamability and foam stability were assessed using an HPHT foam analyzer at 100 °C. The results demonstrate that liquid phase chemistry, experimental conditions, and gas fraction significantly impact foam viscosity. Viscoelastic surfactants achieved a peak foam viscosity of 0.183 Pa·s at a shear rate of 100 1/s and a 70% foam quality, surpassing previous records. At lower foam qualities (≤50%), pressure had a negligible effect on foam viscosity, whereas at higher qualities, it increased viscosity by over 30%. While a slight increase in viscosity was observed with foam qualities between 40% and 60%, a significant enhancement was noted at 65% foam quality. The addition of polymers did not improve foam viscosity. The generation of viscous and stable foams is crucial for effective proppant transport and fracture induction. However, maintaining the thermal stability of CO_2_ foams with minimal additives remains a significant challenge in the industry. This laboratory study provides valuable insights into the development of stable CO_2_ foams for stimulating high-temperature wells.

## 1. Introduction

Foamed fluids consist primarily of a gaseous phase with a dispersion aqueous phase. Foamed fluids minimize water consumption in hydraulic fracturing operations up to 90%, minimize formation damage to high-content clay formations, energize depleted reservoirs, and provide fast cleaning as liquid flows back after depressurization [1]. Additionally, foamed fluids are characterized by excellent proppant transportation and placement due to their high viscosity. Also, they can deliver the proppant deep into the formation and control the geometry of the fractures [2]. The ability of foamed fluids to maintain their flow properties (rheology) and withstand high temperatures (thermal stability) is crucial for successful hydraulic fracturing operations. These characteristics directly influence the efficiency of fluid flow through the created fractures (conductivity) and the overall shape of the fractures [3]. Therefore, the correct measurement of foam viscosity and stability is essential to accomplish a successful hydraulic fracturing treatment.

Foamed fluids exhibit non-Newtonian behavior, characterized by a shear-rate-dependent viscosity. Sun et al. [4] investigated the rheological characteristics of a viscoelastic CO_2_ foam stabilized by 3.8 wt% 18-alkyl trimethylammonium bromide in a large-scale flow loop rheometer under high-pressure conditions (7.1 MPa). A power law model with a strong dependence on temperature and pressure can represent the rheology.

Moreover, Li et al. [5] studied the rheology of CO_2_ foam under several shear rates (100 to 5000 1/s), foam qualities (10 to 70%), and temperatures (35 to 60 °C). The results show that foam viscosity decreased with shear rates, showing a power law model with shear thinning properties; nevertheless, the foam viscosity reached stable values at shear rates higher than 3000 1/s, showing Newtonian behavior. Most of the literature reviews demonstrated that the foamed fluids are described by the power law model [6,7,8,9,10,11].

Foam rheology is strongly affected by several parameters: surfactant type, temperature, pressure, gas–liquid ratio, salinity, and shear rates. Surfactants are used to stabilize and facilitate the generation of bubble foam. At high temperatures (>150 °C), surfactants, especially conventional surfactants, degrade thermally, causing loss of viscosity, and, therefore, premature proppant retention and treatment inefficiency [12]. Numerous studies have explored the flow behavior (rheology) and longevity (stability) of carbon dioxide foam. However, most of the experimental works were carried out at a temperature below 90 °C. Triton X-100 (TX-100) is unaffected by salt content. However, the foam’s resistance to flow, or viscosity, significantly dropped by 20% when the temperature increased up to 90 °C [13]. Furthermore, Sodium dodecyl sulfate (SDS) is an anionic surfactant and cannot produce CO_2_ foam with saline water. It generated a very low foam viscosity of 0.001 Pa·s at low shear rates when the salinity increased to 32,000 ppm [14]. Alpha olefin sulfonates (AOS) are negatively affected by salinity, and they produce very low foam viscosity [15,16]. Switchable amine-based surfactants such as Duomeen TTM, Ethomeen C12, and Ethoduomeen T13 showed excellent performance at high temperatures and salinity [17,18,19]. However, they are only appropriate for low-pH applications such as acid fracturing or matrix acidizing. Previously, Da et al. [20] evaluated the rheological properties of CO_2_ foam stabilized by Duomeen TTM high-temperature (120 °C) conditions in 22% total dissolved solids (TDS) brine. Their findings demonstrated the formation of a thermally stable CO_2_ foam with a viscosity of 0.035 Pa·s. A similar observation was found by Zhang et al. [21].

Recently, viscoelastic surfactants (VES) have attracted special interest due to their compatibility with several chemicals, thermal stability at high temperatures, and salinity tolerance [22,23]. Additionally, it exhibited gel-like characteristics, demonstrating high elasticity, a thickened appearance, and foaming agent properties.

Niu et al. [24] investigated the role of viscoelastic surfactant (erucyl dimethyl amidopropyl betaine, EAB) to stabilize CO_2_ foam at 90 °C, the half-life could reach above 90 min at 70,000 mg/L NaCl or CaCl_2_. Additionally, Alzobaidi et al. [25] studied the rheological properties of CO_2_ foam at 120 °C utilizing zwitterionic amidopropylcarbobetaines in various synthetic water. The CO_2_ foam viscosity of 0.1 Pa·s was achieved at foam quality of 99%, 20.684 MPa, and a 200 1/s shear rate in 2% KCl. Additionally, Alarawi et al. [26] found that 6 wt% of Armovis VES produced a high N_2_ foam viscosity of 0.1 Pa·s at 300 1/s shear rates under harsh reservoir conditions (10.34 MPa, 150 °C).

Foam viscosity is considered a major concern in the hydraulic fracturing process. To address this concern and to enhance foam viscosity, several polymers were utilized, such as polyacrylamide, associative polyacrylamide (super-pusher), partially hydrolyzed polyacrylamide (HPAM), modified guar (CMHPG), guar, and xanthan gum. Al-Darweesh et al. [27] investigated the effect of associative polyacrylamide polymers (super-pusher SAV 522) on the stability and viscosity of CO_2_ foam at 6.89 MPa and 100 °C. Adding 0.5 wt% of super-pusher SAV 522 to a wt% Armovis VES solution significantly increased the foam’s properties. The foam became significantly thicker and more resistant to breaking down (12 times more stable). Similarly, Ahmed et al. [28] found that 0.5 wt% of super-pusher B192 increased the CO_2_ foam viscosity by almost 66.7% at a shear rate of 10 1/s, 80% foam quality, 10.34 MPa, and 80 °C. However, Verma et al. [29] found that the 0.2 wt% of guar polymer improved the stability of foam only by 5 min at 90 °C.

There is a noticeable gap in the existing literature regarding the determination of CO_2_ foam viscosity at high temperatures (>90 °C). The purpose of this paper is to investigate the maximum viscosity that could be achieved using VES at high temperatures. To the best of our knowledge, the thermal stability of CO_2_ foam viscosity has not yet been investigated. Therefore, this work presents the thermal stability of CO_2_ foam viscosity at 150 °C using an HPHT foam rheometer. Additionally, this paper presents the effect of surfactant concentration (3 to 9 wt%), salinity (57,000 to 213,000 ppm), foam quality (40 to 75%), pressure (6.89 to 20.68 MPa), and additives on foam viscosity at high temperature. Furthermore, a specialized HPHT foam analyzer was employed to assess foam stability, formation, and bubble structure under elevated temperature conditions (100 °C). Additionally, this study explores the relationship between foam microstructure and the resistance of CO_2_ foam to flow.

## 2. Results and Discussion

### 2.1. Viscoelastic Surfactants for CO_2_ Foam

**Figure 1:**
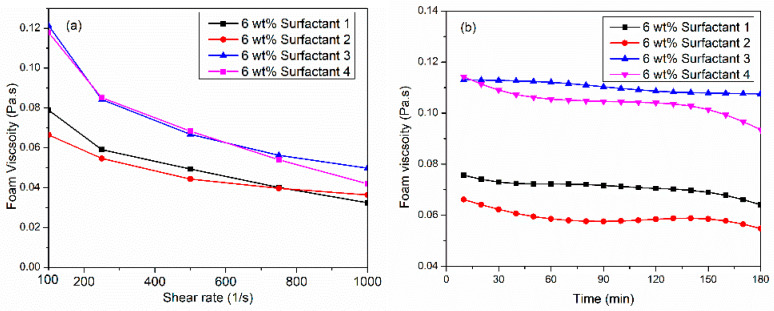
(**a**) Comparative performance of Viscoelastic surfactants for CO_2_ foam viscosity at 150 °C, 6.89 MPa, and 70%, (**b**) thermal foam viscosity at 100 1/s shear rate.

Four viscoelastic surfactants (VES) were assessed for their ability to generate high-viscosity CO_2_ foam under high-pressure (6.89 MPa) and high-temperature (150 °C) conditions. Surfactant solutions were prepared at a concentration of 6 wt% in synthetic seawater (TDS = 57,000 ppm). Foam viscosity was measured across a shear rate range of 100 to 1000 1/s while maintaining a constant foam quality of 70%. As shown in Figure 1a, all VES systems exhibited non-Newtonian shear-thinning behavior, wherein the fluid becomes less viscous under increasing applied force. This rheological response is attributed to foam bubble deformation and structural breakdown. Surfactants 3 and 4 demonstrated superior performance, achieving a maximum foam viscosity of 0.11 Pa·s at a shear rate of 100 1/s.

In contrast, surfactant 1 and surfactant 2 produced foam viscosities of 0.065 to 0.08 Pa·s at 100 1/s. Figure 1b presents the dynamic stability of foam viscosity at a constant shear rate of 100 1/s for 3 h at 150 °C, 6.89 MPa, and foam quality of 70%. The surfactant 4 system initially generated the highest viscosity, but it dropped by 0.03 Pa·s after 180 min. The results indicated that surfactant 3 has the potential to stabilize CO_2_ foam viscosity at high temperatures since the viscosity was only reduced by 0.005 Pa·s after 3 h of foam generated. Nevertheless, the thermal viscosity of surfactant 1, surfactant 2, and surfactant 4 displayed different behavior. The viscosity reduction was obvious for the first 30 min. Then, it remained stable for 2 h, and later, it decreased again. We hypothesize that surfactant 3’s low interfacial tension facilitated increased the brine–CO_2_ interfacial area. This led to accelerated transfer and adsorption of surfactant micelles, resulting in the formation of a higher density of smaller, more uniformly distributed bubbles, ultimately contributing to the observed higher foam viscosity. The achievement of high and stable CO_2_ foam viscosity, utilizing surfactant 3 (110 mPa·s at 100 1/s) in this study holds significant potential for providing excellent proppant carrying and suspension capacity. The literature suggests that CO_2_ foams with viscosities exceeding 50 mPa·s at a shear rate of 170 1/s have been shown to effectively suspend proppants for extended durations (90 min) [30,31].

**Figure 2:**
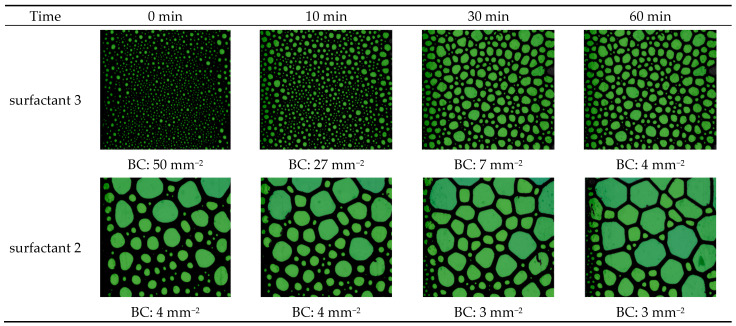
The arrangement and configuration of gas bubbles within the CO_2_ foam at 100 °C and 6.89 MPa using two viscoelastic systems, surfactant 3 and surfactant 2.

An HPHT foam analyzer was utilized at 100 °C, 6.89 MPa to investigate the role of foam structure on foam viscosity. Two viscoelastic systems were utilized: the highest foam viscosity (surfactant 3) and the lowest system (surfactant 2). Figure 2 exhibits the structure of CO_2_ foam at different times: 0, 10, 30, and 60 min. The results indicated that the surfactant 3 system initially generated the highest number of foam bubbles (BC), 50 mm^−2^, with a fine homogenous texture. However, surfactant 2 produced a very low number of foam bubbles and a coarse texture with non-uniform distribution, which resulted in lower viscosity. Bubble formation is influenced by the diffusion and adsorption kinetics of surfactant molecules at the CO_2_/liquid interface. During the foaming process, we believed that the micelles of surfactant 3 moved faster to the interface to generate other bubbles, while the larger bubbles broke into multi-small bubbles.

Additionally, the surfactant 2 system has higher foam stability than the surfactant 3 system since the bubble count was reduced by 46% for surfactant 3 after 10 min, and it almost remained constant for surfactant 2. Therefore, generating a higher number of fine-texture bubbles resulted in higher viscosity.

### 2.2. Effect of Surfactant Concentration on CO_2_ Foam Viscosity

**Figure 3:**
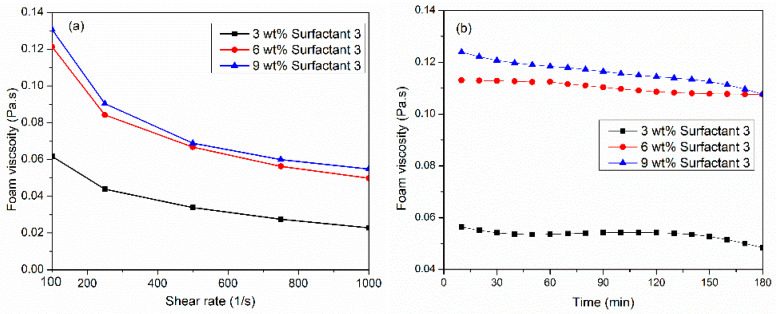
(**a**) Effect of surfactant concentration on CO_2_ viscosity at 150 °C, 6.89 MPa, and 70%, (**b**) thermal foam viscosity at 100 1/s shear rate using three concentrations of surfactant 3 system.

This part investigates the effect of surfactant concentration on CO_2_ foam viscosity at 150 °C and 6.89 MPa. Figure 3a illustrates the effect of three concentrations (3, 6, 9 wt%) of the surfactant 3 system at different shear rates and foam quality of 70%. At a concentration of 3 wt%, relatively low foam viscosity was attained at 0.06 Pa·s at a 100 1/s shear rate. When surfactant load increased to 6 wt%, foam increased almost by 100%. However, surfactant concentration from 6 wt% to 9 wt% had no major increase in CO_2_ foam viscosity. Figure 3b studied the thermal stability of surfactant 3 systems using three concentrations at a constant shear rate of 100 1/s for 180 min. Interestingly, 6 wt% of the surfactant 3 system generated the most stable viscosity as compared with 3 wt% or 9 wt%. When surfactant 3 concentration increased from 6 to 9 wt%, the viscosity after three hours remained unchanged, but it decreased with time. Technically, the initial bubbling process produces a coarse foam comprising large bubbles, which are subsequently ruptured into smaller bubbles to generate the final foam [32]. During the shearing, bubbles were broken up, creating a new gas/liquid interface with sufficient micelles due to the faster adsorption rate. This caused the formation of several new fine-textured bubbles. Increasing the surfactant concentration reduced the CO_2_–brine interface, through which the micelles traveled, and they were adsorbed faster at the interface to generate smaller and more stable bubbles and therefore increased the bubble density, which is considered the main contributor to foam viscosity. Additionally, this resulted in a thicker and more elastic liquid film, which provided greater resistance.

### 2.3. Water Chemistry Role on CO_2_ Foam Viscosity

**Figure 4:**
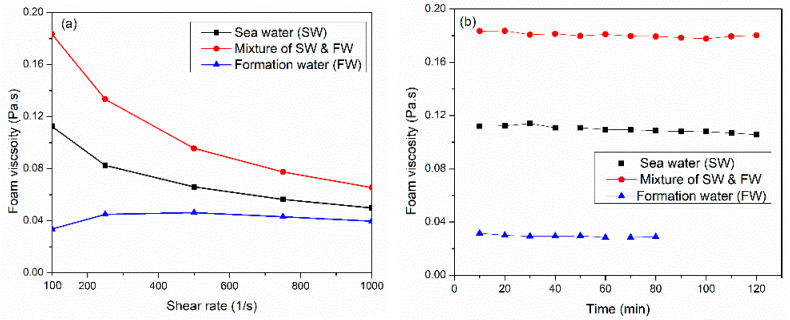
(**a**) Water chemistry affects CO_2_ foam viscosity at 150 °C, 6.89 MPa, and 70%, and (**b**) thermal foam viscosity at 100 1/s shear rate using different water salinities.

**Figure 5:**
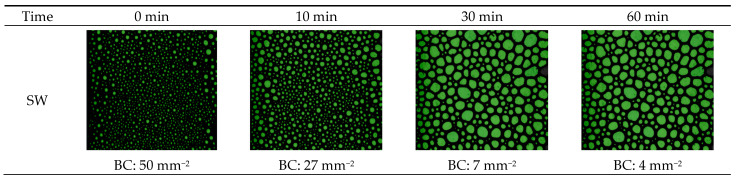

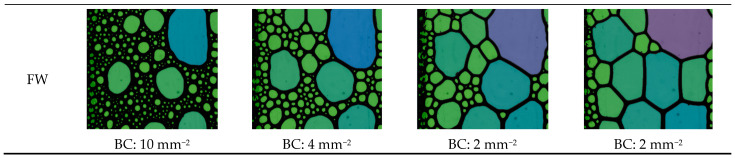
The arrangement and configuration of gas bubbles within the CO_2_ foam at 100 °C and 6.89 MPa for two systems: synthetically prepared seawater (SW) and formation water (FW).

Water chemistry plays a crucial role in foam viscosity; it can directly impact the stability and the performance of foam in the fracturing process and thus affect the proppant transportation and placement. Therefore, understanding and optimizing water chemistry ensures the effective performance of foam in hydraulic fracturing. Three salinities were investigated (57,000, 148,500, and 230,000 ppm) at 150 °C and 6.89 MPa. The liquid phase comprised a 6 wt% solution of surfactant 3 in varying saline water concentrations. As illustrated in Figure 4a, high-salinity (~213,000 ppm) water representing high-salinity formation water obtained low-viscosity, thickening behavior at low shear rates, and a constant viscosity at higher shear rates. As the salinity increases, the adsorption rate at the interface increases due to the reduction in the electrostatic repulsion force, which allowed surfactant micelles to move into the interface to generate fine-texture bubbles. However, increasing the salinity to 213,000 ppm hindered the transfer and adsorption of micelles at the interface due to the weakening of the hydration shell around the surfactant (see Figure 5). Therefore, the pressure difference between the bubble film surface and the plateau boundary with a high curvature radius also increased. This behavior accelerated the film thinning process until the film ruptured [33].

Nevertheless, the mixture of both synthetically prepared seawater and formation water (~108,500 ppm) generates the maximum foam viscosity of 0.183 Pa·s at a 100 1/s shear rate. Figure 4b displays the thermal stability of CO_2_ foam viscosity, utilizing different water salinities at a shear rate of 100 1/s. Synthetically prepared water representing formation water generated unstable foam; after 80 min, the foam collapsed and separated.

The effect of water salinity on the foam structure was studied. Two water types were investigated: synthetically prepared seawater and formation water. Figure 5 presents the structure of CO_2_ foam at 100 °C and 6.89 MPa at given time intervals: 0, 10, 30, and 60 min. The findings suggest that a thicker foam is associated with a greater number of smaller, evenly distributed bubbles. The seawater-based system produced an exceptionally high concentration of bubbles, with 50 mm^−2^, while the formation water system generated fewer bubbles per area of 10 mm^−2^, coarse texture, and heterogeneous bubble distribution. This could be attributed to the chemistry of the fluid where the higher salt concentrations shielded the micelles and hindered their travel to the interface, which resulted in poor foamability and, thus, low viscosity. Moreover, the SW system generated higher stable CO_2_ foam since the bubble count was reduced by 46% within 10 min, while 60% for the FW system.

### 2.4. The Effect of Polymer on CO_2_ Foam Viscosity

**Figure 6:**
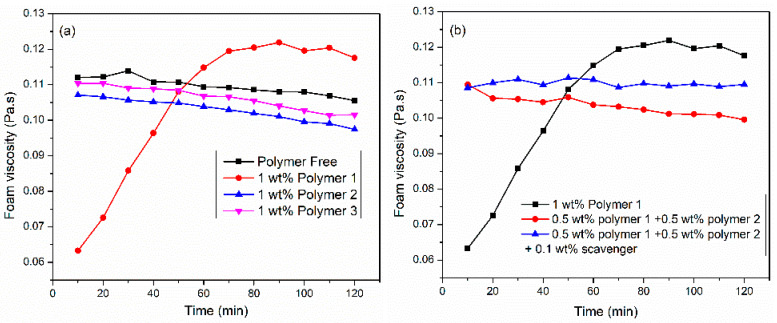
(**a**) Effect of polymers and (**b**) polymer–mixture on VES CO_2_ foam viscosity at 150 °C, 6.89 MPa, 100 1/s, and 70%.

The polymer was utilized to enhance foam viscosity by increasing the thin film thickness and reducing the mobility of foam bubbles. In this Section, three polymers were investigated: modified guar-based (polymer 1), polymer 2, and polymer 3. The liquid formulation was composed of SW, 6 wt% surfactant 3, and 1 wt% polymer. Figure 6a illustrates the influence of polymer addition on CO_2_ foam viscosity under conditions of 150 °C, 6.89 MPa, 100 1/s shear rate, and 70% foam quality. Polymer 1 exhibited slow hydration kinetics when added to the surfactant solution. A gradual increase in foam viscosity was observed over time, reaching 0.12 Pa·s after 60 min. Faster hydration was observed in polymer 2 and polymer 3.

Nevertheless, the addition of 1 wt% polymer to viscoelastic surfactant (6 wt% surfactant 3) did not increase foam viscosity. As shown in Figure 6b, the polymer mixture system resulted in faster hydration; nevertheless, there was no increase in foam viscosity. The structure and interaction of bubbles strongly influence foam viscosity. We suspect that the polymer did not interact with the gas–liquid interface. Nevertheless, the literature review [12] showed that the polymers decomposed at 150 °C due to the attack of oxygen in the backbone of the polymer. Therefore, 0.1 wt% oxygen scavenger was added to prevent the oxidation and degradation of polymers. The results suggested that the addition of a scavenger presented the most stable foam without any increase in foam viscosity.

### 2.5. The Role of Elevated Pressure and Foam Quality on CO_2_ Foam Viscosity

**Figure 7:**
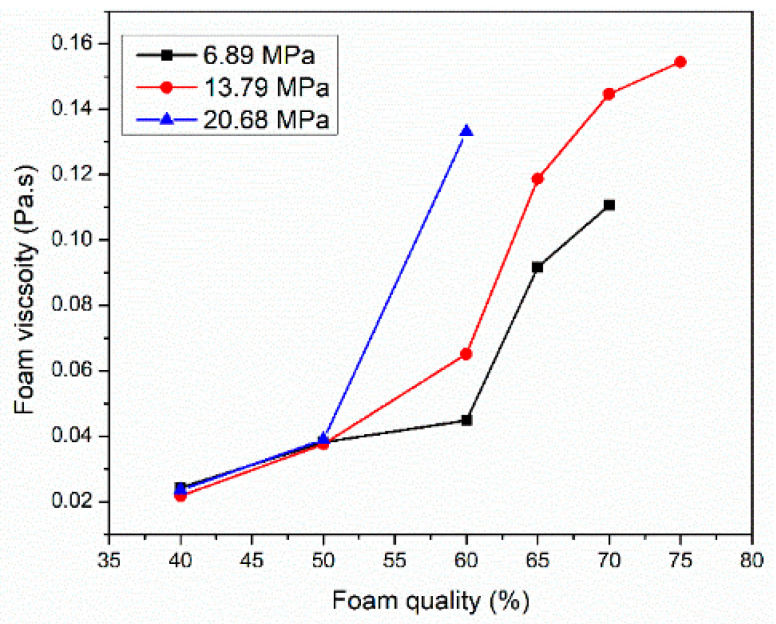
The role of elevated pressure and foam quality on CO_2_ foam viscosity at 150 °C and a shear rate of 100 1/s.

Figure 7 displays the effect of elevated pressures 6.89, 13.79, and 20.68 MPa with different ranges of foam qualities on the CO_2_ foamed viscosity at 150 °C and a shear rate of 100 1/s. The liquid solution contained 6 wt% of surfactant 3 dissolved into SW. At low foam qualities of 40 and 50%, pressure did not affect the foam viscosity. In this regime, the foam viscosity is largely independent of pressure because the liquid phase dictates the viscosity and the liquid phases’ properties do not change significantly with pressure. However, the foam viscosity increased by 30% when the pressure was raised to 13.79 MPa for higher foam qualities. As pressure increases, CO_2_-brine surface tension decreases, leading to an increase in foam dispersibility, forming a uniform and finely bubbles and therefore, higher foam viscosity [34]. While some literature suggests that pressure has a negligible impact on foam viscosity [31], our findings indicate a positive correlation, especially in the presence of viscoelastic surfactants. We hypothesize that these surfactants may increase the solubility of CO_2_ in the brine phase at higher pressures, further reducing interfacial tension and promoting the formation of denser foams. The foam viscosity increased gradually when the foam quality increased from 40 to 50%. However, a sharp increase was observed at a foam quality of 65%. As foam quality increases, more bubbles are generated with strong interaction, creating a more structured network, which increases the foam’s resistance to deformation and flow. Similar results were found by Wang et al. [35].

**Figure 8:**
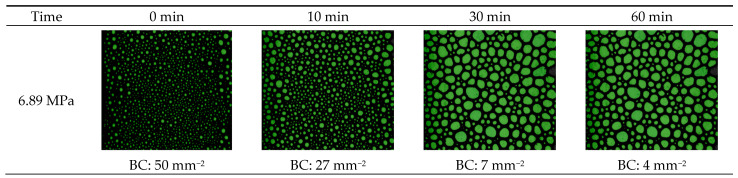

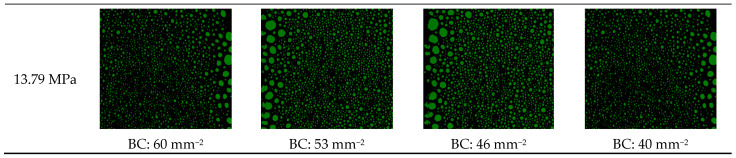
The arrangement and configuration of gas bubbles within the CO_2_ at elevated pressure.

Figure 8 presents the effect of pressure on foam structure at pressures of 6.89 MPa and 13.79 MPa at given time intervals of 0, 10, 30, and 60 min. The outcomes indicated that the higher pressure generated a high number of bubbles with uniform fine texture and, thus, higher viscosity. Additionally, high pressure leads to higher foam stability since the bubble count was reduced by 46% after 10 min for 6.89 MPa; however, it was only by 12% for higher pressure of 13.79 MPa.

### 2.6. The Stability of CO_2_ Foam

**Figure 9:**
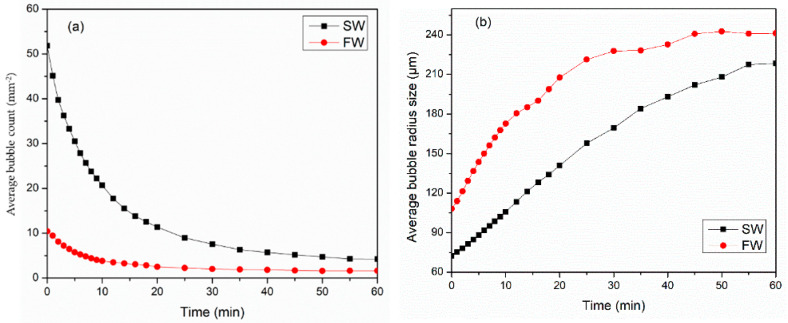
Water chemistry impact on the (**a**) average bubble count (mm^−2^) and (**b**) average bubble radius (μm) at 100 °C and 6.89 MPa.

Foam is unstable, and as a result of time, the two-phase mixture tends to separate into liquid and gas phases. Three main mechanisms govern the stability of foam: liquid drainage, bubble coarsening, and bubble coalescence. Therefore, this section studies the behavior of foam during static conditions using an HPHT foam analyzer. Figure 9 presents a summary of how the salt content in water affects the ability to create (foamability) and maintain (stability) carbon dioxide foam under high-temperature and -pressure conditions. Surfactant 3 is strongly dependent on the water chemistry. The SW system showed higher foamability as compared to the FW system; it produced 50 bubbles/mm^2^, while FW generated only 10 bubbles/mm^2^. Figure 9a presents the decay behavior of bubble count per area for SW and FW systems. For the first 15 min, both systems observed a sharp reduction in bubble count, followed by a gentle decrease. The sharp decrease was due to the immediate collapse of the upper layers and the disappearance of the tiny bubbles. For the SW system, the bubble count was reduced by 50% after 7 min of foam generation. The bubble count half-life for the FW system was 6 min, as shown in Table 1.

Figure 9b illustrates the bubble coarsening rate of CO_2_ foam with time for SW and FW systems at 100 °C and 6.89 MPa for 60 min. For both systems, foam bubble size immediately grows after generation. Bubble coarsening takes place due to the gas diffusion from smaller bubbles to larger ones, which subsequently results in the vanishing of tiny bubbles. Nevertheless, the growth of a bubble is governed mainly by the permeability of the bubble film, gas solubility, and diffusivity [36]. The bubble coarsening behavior for the SW system was linear, while for the FW systems, it increased significantly within 20 min, and then it gradually grew for 40 min. The bubble coarsening rate was 10 min for the SW system and 7 min for the FW system.

**Figure 10:**
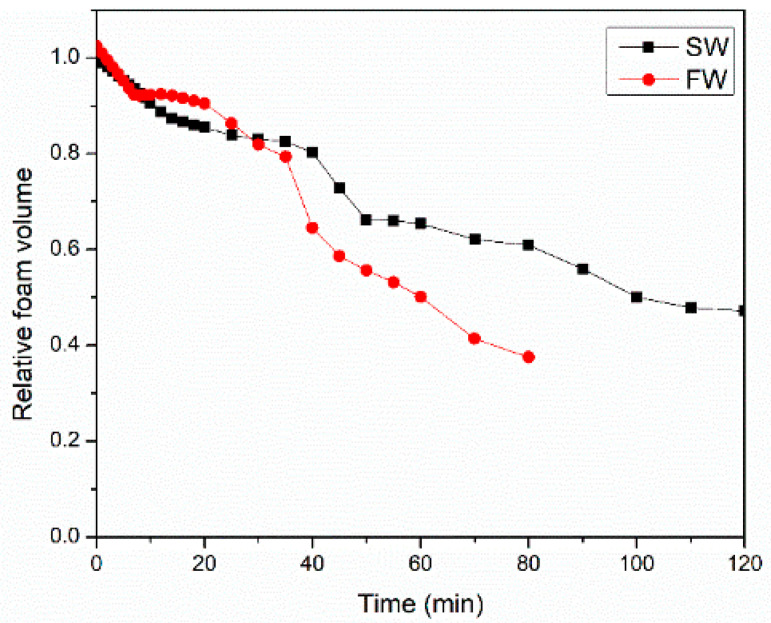
Water chemistry impacts the CO_2_ foam volume decay process at 100 °C and 6.89 MPa.

Figure 10 shows the effect of surfactant solution salinity on the evolution of CO_2_ foam volume 100 °C and 6.89 MPa. The newly formed foam rapidly collapsed due to destabilizing factors. Seawater-based systems exhibited significantly greater foam stability compared to formation water systems. This enhanced stability is attributed to seawater’s ability to hinder the dissolution and penetration of CO_2_ into the liquid film, thereby reducing bubble deformation and prolonging foam life. Additionally, the liquid drainage rate for SW was slower than the FW system, which kept the liquid film stronger for a longer time. The foam half-life for SW and FW were 80 min and 60 min, respectively.

## 3. Conclusions

This paper investigated the rheology of CO_2_ foamed fracturing fluid utilizing commercial surfactants at high temperatures (150 °C) through the use of an HPHT foam rheometer. Seawater was used as a replacement for fresh water. The effect of several parameters on foam viscosity, such as pressure, CO_2_ fraction, surfactant concentration, and water chemistry, was investigated. Additionally, this paper found a strong relationship between foam viscosity and bubble texture using an HPHT foam analyzer at 100 °C. The following conclusions can be drawn:

- Surfactant 3 exhibited the highest viscosity at 150 °C and 6.89 MPa because it produced a higher number of fine-texture bubbles with uniform distribution. The results suggested that smaller bubble sizes can produce higher foam viscosity.
- 6 wt% surfactant 3 has the potential to suspend and deliver the proppant into fracture since it attained a high CO_2_ foam viscosity of 0.11 Pa·s at a 100 1/s shear rate and stabilized the foam viscosity almost unchanged for 180 min.
- Increasing the surfactant 3 concentration above 6 wt% or adding polymers did not increase CO_2_ foam viscosity.
- Water chemistry has a significant impact on foam viscosity. Once the salinity increased to 108,720 ppm, it increased to 0.183 Pa·s at 100 1/s; nevertheless, it reduced to 0.035 Pa·s at 100 1/s when the salinity increased to 213,734 ppm.
- This study shows that the foam viscosity increased almost by 30% when the pressure increased to 13.79 MPa (supercritical condition: 7.38 MPa, 31 °C). Hence, 6 wt% surfactant 3 was capable of generating CO_2_ foam at supercritical conditions.
- A comprehensive investigation of foam stability, microstructure, and bubble number at 100 °C provided insights into foam collapse mechanisms. HPHT foam analyzer data established a correlation between bubble size distribution and foam viscosity. Additionally, the FW system shows poor foamability and poor stability; however, the foam half-life extended to 80 min for the SW system.

## 4. Materials and Methods

### 4.1. Chemicals and Materials

In this study, four commercial viscoelastic surfactants were used to generate CO_2_ foam at 150 °C. Three polymers were utilized to enhance foam viscosity. An oxygen scavenger was used to prevent the decomposition of polymers at high temperatures. The details of the chemicals utilized are presented in Table 2.

The study encompassed five different types of salts: sodium chloride (NaCl) with assay of ≥99.5%, calcium chloride (CaCl_2_) with assay of 99–102%, magnesium chloride (MgCl_2_) with assay of 99–101%, sodium sulfate (Na_2_SO_4_) with assay of ≥99.5%, and sodium bicarbonate (NaHCO_3_) with assay of 99–101% to prepare the synthetically prepared seawater and formation water by varying their concentrations. The chemical composition of synthetically prepared seawater and formation water are illustrated in Table 3. The surfactant solution is usually prepared by dissolving the surfactant into saline water. Then, the solution was mixed for 24 h at room temperature using a magnetic starrier plate at 500 revolutions per minute (rev/min). However, the solution exhibited gel-like characteristics, demonstrating high elasticity and a thickened appearance.

### 4.2. HPHT Foam Rheometer

**Figure 11:**
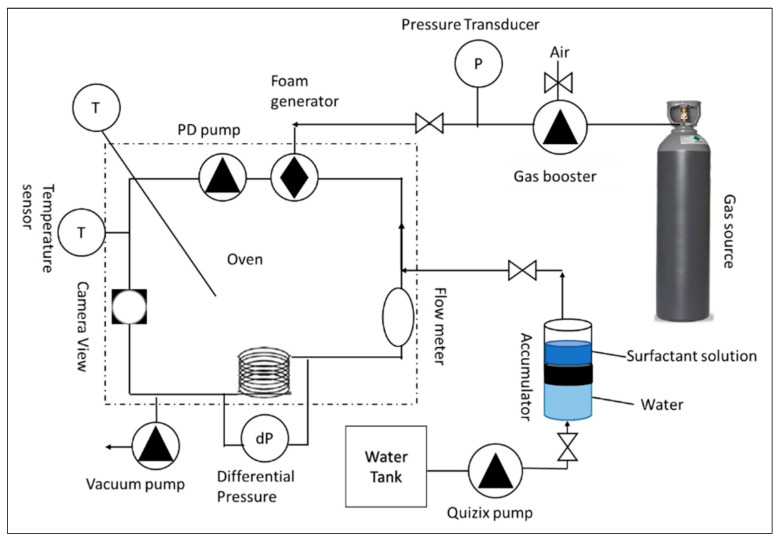
Simplified representative of high-pressure, high-temperature foam rheometer.

HPHT foam rheometer is implemented to determine the foam’s viscosity, density, and quality across a range of shear rates. As displayed in Figure 11, the major components of this equipment are a flow loop, oven, flow meter, densitometer to evaluate liquid and foam densities, view cell to visualize the generation of foam, transducers to measure the pressure differences, and circulation pump to shear and generate foam. To maintain a consistent fluid temperature throughout the experiment, all components were enclosed within an oven.

The experimental procedure commenced by evacuating the system to remove residual liquid and gas. Subsequently, the accumulator was charged with the liquid solution. A volume of 130 mL of this solution was then transferred to the flow loop via the Quizix pump. Upon complete liquid transfer, the oven temperature was set to 70 °C to avoid the evaporation of surfactant solution in the flow loop. Since the expansion of the liquid can burst the tubing. Once thermal equilibrium was achieved, gas was introduced into the loop using the booster pump until the target pressure was attained. Foam generation was initiated by applying a shear rate of 500 1/s until visual homogeneity was confirmed using a view cell camera. Subsequently, the oven temperature was elevated to the desired test temperature of 150 °C. Additional gas was injected to achieve the requisite test pressure. Manual adjustment of gas and liquid volumes ensured a consistent foam quality of 70% throughout the experiments. Optimal test conditions, encompassing temperature, pressure, and foam quality, were established prior to implementing an automated data acquisition protocol. This protocol recorded data at 15–40 min intervals while incrementally increasing the shear rate from 100 to 1000 1/s.

The apparent foam viscosity is determined through the following formula:

(1) $$\mu_{app}=\frac{1000\tau }{\dot{\gamma }}=\frac{1000\left(D\frac{∆P}{4L}\right)}{\left(\frac{8U}{D}\right)}$$

where the $\mu_{app}$ is apparent viscosity of foam (cp), the shear stress (τ) was obtained via the differential transducers positioned at the inlet and outflow of the coil loop (pascal), The shear rate $(\dot{\gamma }$) was calculated from the built-in mass flow meter (1/s), L is the length of the loop (cm), ΔP is the pressure difference (pascal), U is the velocity of foam inside the loop (cm/s), and D is the loop diameter (cm). 

The foam quality is integrated using the mass balance equation as the following:

(2) $$FQ=\frac{\rho_{l}-\rho_{foam}}{\rho_{l}-\rho_{gas}}$$

The densities of surfactant solution ($\rho_{l}$) and foam ($\rho_{foam}$) were determined experimentally using a densitometer integrated within the mass flow meter. The density of CO_2_ ($\rho_{gas}$) was calculated using the REFPROP database and the Span–Wagner Equation of State. All experiments were conducted at a temperature of 150 °C, pressures of 6.89 to 20.68 MPa, and foam qualities of 40 to 70%. The test matrix of experiments is shown in Table 4. Firstly, the performance of several viscoelastic surfactants was investigated at 6.89 MPa, with foam quality of 70% and shear rates ranging from 100 to 1000 1/s. Then, the critical concentration of the optimum surfactants (surfactant 3) was studied. Later, the role of water chemistry was determined utilizing three water salinities. Also, the effect of additives such as polymers and gel stabilizers on CO_2_ foam viscosity was conducted utilizing 1 wt% of different polymers. Finally, the influence of experimental conditions such as pressure and foam quality was investigated.

### 4.3. HPHT Foam Analyzer

**Figure 12:**
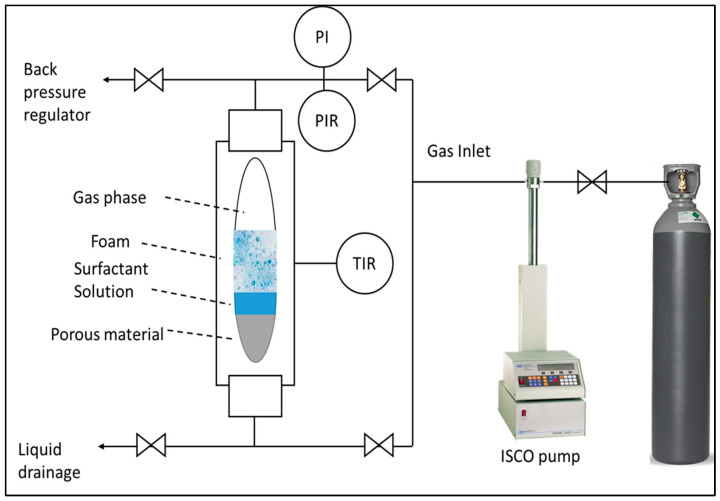
Simplified representative of high-pressure, high-temperature foam analyzer.

An HPHT foam analyzer was employed to characterize foam structure, stability, and foamability under conditions of 100 °C and 6.89 MPa. As depicted in Figure 12, the apparatus comprised a high-pressure view cell equipped with an electric heat jacket, a camera, a transmitted light source, a prism, a temperature controller, and an ISCO pump. The experimental protocol commenced by filling the view cell with surfactant solution and heating it to 100 °C. Subsequently, 300 mL of the liquid phase was drained, and the system pressurized to 6.89 MPa. Foam generation was initiated by injecting CO_2_ at a constant flow rate of 50 mL/min through porous media at the base of the view cell. A backpressure valve regulates system pressure during the foaming process. The foam generation and decay process was captured on video and subsequently analyzed using ADVANCE software (version 1.16).

The foam height function was predicated on the assumption of light transparency through both gas and liquid phases. However, the opaque nature of foam impedes light transmission, enabling the photodetector to discriminate between liquid, foam, and gas interfaces. Foam structure visualization was achieved through a prism-based optical system incorporating transmitted light and a camera. Positioned perpendicular to the prism above the view cell, the system exploited the refractive index differential between media to capture bubble morphology. The presence of a thin liquid film at the bubble-prism interface facilitated partial light diffraction, permitting limited penetration into the foam. Conversely, complete light diffraction occurred at the gas–prism interface.

Table 5 outlines the experimental methodology employed using an HPHT foam analyzer at 100 °C. Tests 1 and 2 focused on elucidating the correlation between foam microstructure and CO_2_ foam viscosity. Test 3 investigated the influence of water chemistry on foam stability and structure in the presence of CO_2_. Test 4 examined the impact of CO_2_ pressure on bubble morphology and foam stability.

## Figures and Tables

**Table 1:** Water chemistry affects the foamability and stability of CO_2_ foam at 100 °C and 6.89 MPa.

Test	Initial Bubble Count per Area (mm^−2^)	Initial Bubble Radius Size (μm)	Bubble Count Half-Life (min)	Bubble Coarsening Rate (min)	Foam Half-Life (min)
SW	50	75	7	10	80
FW	10	105	6	7	60

**Table 2:** The details of used chemicals.

Chemical Code	Chemical Name
Surfactant 1	Erucamidopropyl Hydroxypropylsultain
Surfactant 2	derived from Ammonium Quaternary compound and propanol
Surfactant 3	
Surfactant 4	
Polymer 1	Carboxymethyl Hydroxypropyl Guar Gum (CMHPG), Molecular weight of 20 million Dalton.
Polymer 2	Made of 50–65% Acrylamide (AM) and 35–50% N-vinylprrolidone (NVP).
Polymer 3	Made of 25–45% Acrylamide (AM), 20–25% 2-Acrylamido-2-methylpropane sulfonic (ATBS), and 35–50% N-vinylprrolidone (NVP).
Oxygen scavenger	sodium thiosulfate

**Table 3:** The salt compositions of synthetic water.

	Composition	Seawater (SW) (g/L)	Formation Water (FW) (g/L)
1	NaCl	41.2	150.5
2	MgCl_2_·6H_2_O	17.6	20.4
3	NaHCO_3_	0.17	0.49
4	Na_2_SO_4_	6.33	0.52
5	CaCl_2_·2H_2_O	2.39	69.8
6	TDS	57.7	213.7

**Table 4:** The experimental design for HPHT foam rheometer.

Investigation	Surfactant Type	Surfactant Concentration (wt%)	Water Type	Polymer (wt%)	Foam Quality (%)	Pressure (MPa)
Comparative Performance	-Surfactant 1 -Surfactant 2 -Surfactant 3 -Surfactant 4	6	SW		70	6.89
Surfactant concentration	Surfactant 3	−3 −6 −9	SW		70	6.89
Water chemistry	Surfactant 3	6	-SW -FW -SW+FW		70	6.89
Polymer	Surfactant 3	6	SW	-polymer 1 -polymer 2 -polymer 3	70	6.89
Foam Quality	Surfactant 3	6	SW		−40 to 7	6.89
Pressure	Surfactant 3	6	SW		7	−6.89 −13.79 −20.68

**Table 5:** Experimental design for HPHT foam analyzer at 100 °C.

Test No.	Surfactant Type (wt%)	Water Type	Pressure (MPa)
1	6 wt% surfactant 3	SW	6.89
2	6 wt% surfactant 2	SW	6.89
3	6 wt% surfactant 3	FW	6.89
4	6 wt% surfactant 3	SW	13.79

## Data Availability

The original contributions presented in this study are included in the article. Further inquiries can be directed to the corresponding author.
